# A Position Statement on Optimizing the Role of Oncoplastic Breast Surgery

**Published:** 2012-08-23

**Authors:** Christoph Andree, Jian Farhadi, Dennis Goossens, Jaume Masia, Isabelle Sarfati, Günter Germann, R. Douglas Macmillan, Michael Scheflan, Hans-Pieter Van Not, Giuseppe Catanuto, Maurizio Bruno Nava

**Affiliations:** ^a^Department of Plastic and Aesthetic Surgery, Sana Hospital Düsseldorf, Germany; ^b^Department of Plastic Surgery, Guy's and St Thomas' Hospital, Westminster Bridge Road, London, United Kingdom; ^c^Ziekenhuis Walcheren, Middelburgsestraat, Zeeland, The Netherlands; ^d^Plastic Surgery Department, Sant Pau University Hospital, Barcelona, Spain; ^e^L-Institute du Sein, Paris, France; ^f^Clinic for Plastic and Reconstructive Surgery, Preventive and Aesthetic Medicine at Heidelberg University Hospital, Heidelberg, Germany; ^g^Nottingham Breast Institute, City Hospital, Nottingham, United Kingdom; ^h^Atidim Medical Center, Tel-Aviv, Israel; ^i^Adriaan de Ruyter Ziekenhuis Goes, Goes, The Netherlands; ^j^Unit of Plastic and Reconstructive Surgery, Fondazione IRCCS, Istituto Nazionale Tumori, Milano, Italy; ^k^Fondazione IRCCS Istituto Nazionale dei Tumori Via Venezian, Milano, Italy

## Abstract

**Objectives:** To propose initiatives and actions that could improve access to and outcomes from oncoplastic breast surgery. **Methods:** The author group met in May 2010 to draft position statements on key unmet needs in oncoplastic breast surgery and how these may be addressed. At a second meeting in December 2010, the statements were voted upon and adjusted as necessary to achieve unanimous agreement. **Results:** It was agreed that every patient undergoing breast cancer surgery should be assessed by an oncoplastic team capable of offering the full range of surgical options. However, currently, not all women are adequately informed about the surgical options available. Furthermore, levels of multidisciplinary working, standards of care, and levels of surgical training in the full range of breast oncoplastic techniques are suboptimal. Institution-specific guidelines relating to the optimal patient pathway, the definition of clinical standards, and improved education in reconstructive surgery are required. Oncoplastic breast surgery should be offered to all patients, within the context of multidisciplinary teams that include accredited surgeons who consult with each other early in the treatment pathway. These teams should be focused on achieving not just positive oncologic outcomes, but also esthetic outcomes in line with patient wishes, to achieve optimal quality of life. **Conclusions:** There is a desire within the surgical community to improve patient outcomes by better incorporating oncoplastic procedures into the treatment pathways for breast cancer. These position statements represent the perspectives of a group of European plastic surgeons on the key elements required to achieve this goal.

The surgical options for breast cancer fall into 2 main categories—mastectomy or breast conserving surgery (BCS). Many new oncoplastic breast surgery (OPBS) techniques have extended the role of BCS and enabled better esthetic outcomes from both BCS and mastectomy.^1^ In recent years, there has been some debate over the definition of “OPBS,” but we believe that *all* breast cancer surgery is oncoplastic. As such, OPBS may be defined as breast cancer surgery focused on optimizing both oncologic and esthetic outcomes, irrespective of the type(s) of surgery performed. All breast cancer surgery should be performed with the principles of OPBS in mind.

Oncoplastic breast surgery is becoming increasingly established in all aspects of the surgical management of breast cancer and can be conducted in a variety of different ways depending, for example, on the original breast size, tumor size, degree of ptosis, use and timing of radiotherapy, patient preference, and characteristics of potential donor sites.^2^ The techniques may involve a combination of implant-based and autologous tissue-based approaches and incorporate many cosmetic surgical techniques, timed according to immediate or delayed protocols based on various factors, including the type of tumor-removal operation performed and the use and extent of adjuvant therapies.^2^

The range of possibilities in breast reconstruction has also been enhanced in recent years by the development of skin-sparing mastectomy techniques. In these procedures, the tumor is removed, with or without preservation of the nipple–areola complex (NAC), and with removal of the underlying glandular tissue, leaving the skin to cover the implant or autologous, transferred tissue.^3^ The results obtained with skin-sparing mastectomies have been good, in terms of both cosmetic and oncologic outcomes.^4^ However, while we anticipate that the use of skin-sparing mastectomies will continue to increase in the future, not all cases will be suitable. Indeed, progress in the surgical treatment of breast cancer has resulted in a wide variety of techniques that may be suitable for a range of different patients and stages of disease.

The use of contralateral surgery is a relatively new concept in breast cancer surgery. Overall breast adjustment – for example, overall reduction with BCS or overall enlargement with implant reconstruction – is often an option that facilitates cancer excision and enhances esthetic outcomes.^5^ For many patients, a contralateral reduction may improve quality of life in conjunction with a simple mastectomy.

## OPTIMIZING ONCOLOGIC AND ESTHETIC OUTCOMES

Although mastectomy is an effective procedure, it can have a negative impact on body image, sense of attractiveness, and sexual function.^6^ Breast conserving surgery, which aims to conserve as much of the undiseased breast tissue as possible, is viewed by many surgeons as the option that most patients prefer, particularly those who are fully informed about all the available choices. However, survey data from a cohort of 125 women, who underwent surgical treatment of breast cancer and who were educated on mastectomy and BCS, showed that 35% opted for mastectomy even though they understood the benefits of BCS.^7^ In the United States, the percentage of patients undergoing mastectomy has risen in recent years.^8^ This may be due to increased use of magnetic resonance imaging, which commonly identifies additional tumor foci in newly diagnosed patients, leading in some cases to a mastectomy that might not have been undertaken otherwise.^9^ It could also be driven by fear of disease recurrence or by the poor esthetic outcomes that are reported by approximately 30% of women after BCS.^10^^,^^11^

For those patients who choose or are recommended that, mastectomy coupled with breast reconstructive surgery offers the dual benefits of confidence in tumor eradication and the restoration of femininity, body image, vitality, and quality of life.^12^^,^^13^ In patients who have undergone breast reconstruction following mastectomy, quality of life scores have been found to be equivalent to those of the general population.^12^ Despite these successes, rates of breast reconstruction are low. In a recent audit of 18 216 patients in the United Kingdom who underwent mastectomy, less than 30% received a breast reconstruction (18.6% immediate reconstruction; 9.5% delayed reconstruction).^14^ It is possible that this poor uptake of reconstructive surgery is driven in part by a failure by some attending physicians to discuss reconstructive options with patients. In addition, institutions vary in the procedures they provide, and this may limit the choices available to patients.^15^ Also, in some instances, there may remain a disconnection of practice and communication between the various specialties involved in managing breast cancer surgery. The aim should be to discuss all options with patients, and to make the full range of surgical procedures available. If this were the case, we would anticipate a rise in the level of breast reconstruction procedures.

Data on the availability and uptake of OPBS techniques in BCS are not available, but it is likely that a similar situation exists. It is also likely that the number of patients opting for oncoplastic breast surgery with mastectomy or BCS would increase if surgical options were adequately discussed with all such patients before they started treatment. Indeed, in the UK audit, women who had undergone mastectomy without reconstruction were asked about the information they had received on reconstruction; only 65% felt that they had received the right amount of information, and 42% felt that a lack of information had contributed to not choosing a reconstruction.^14^ Patients may also benefit from receiving information through a variety of different media, including informational booklets, photographs, and videos.^16^

## BEST PRACTICE IN BREAST CANCER SURGERY

To achieve the best results from the range of techniques available for breast cancer surgery, it is important to maintain a strong multidisciplinary collaboration between the surgical and nonsurgical teams involved. A key requirement for optimal outcomes is that the surgery for removing the tumor or breast is performed in a way that maximizes the chances of a good esthetic result from any concomitant or subsequent reconstructive surgery. This requires that the reconstructive surgeon be involved early in the treatment pathway, at a point where he/she can take part in the decision on the technique to be used to remove the tumor.

While a multidisciplinary collaborative approach is important in optimizing outcomes, it is also essential that reconstructive surgeons (irrespective of their surgical specialty) are specialized and formally trained in the full range of OPBS techniques. Unfortunately, the availability of training in OPBS is currently insufficient, and the techniques that are used vary widely between countries and institutions.^17^ A recent international consensus, arising from a symposium on training in oncoplastic surgery in Portugal, Spain, Brazil, and the UK, reported significant variations in the levels of oncoplastic services in different countries and highlighted a lack of available skills, variable patient information, and differences in levels of access to procedures.^17^

On the basis of aforementioned facts, it is clear that there is a desire within the surgical community to improve patient outcomes by incorporating oncoplastic procedures into treatment pathways for breast cancer, but that progress is hindered by a lack of training, under-education of patients regarding treatment choices, an absence of clearly defined surgical practice standards, and difficulties in forming multidisciplinary treatment teams.

This article contains a series of position statements from a group of plastic surgeons who met to discuss how key unmet needs in reconstructive surgery can be addressed.

## METHODS

The authors of this article (all breast oncoplastic surgeons) met at an Allergan-sponsored working group meeting in Manchester, United Kingdom, in May 2010 to discuss the current situation in European oncoplastic breast surgery. During this meeting, a series of position statements were drafted. These statements defined the group's views on current unmet needs in OPBS and how these needs could be addressed. At a second meeting held in Milan, Italy, in December 2010, the position statements were reviewed and adjusted as necessary, such that each statement had the endorsement of all 11 members of the author group.

## RESULTS: POSITION STATEMENTS FROM THE GROUP

The group agreed that the goal is to have every patient with breast cancer evaluated by an oncoplastic team that is capable of offering the full range of oncoplastic surgical options. On the basis of the available literature, evidence, and personal observations, the group defined the following unmet needs in breast cancer surgery:

- Improved definition of, and alignment between, the various specialties involved in the management of breast cancer patients to ensure a well-planned multidisciplinary approach to treatment.
- Within that multidisciplinary approach, closer cooperation and skill sharing between the surgeons involved in excising the tumor and those involved in breast reconstruction.
- The definition of measurable working practice standards.
- Improved training among surgeons performing reconstructive surgery to ensure that patients are offered all available options for which they are suitable.
- Formal oncoplastic training programs that integrate training between breast oncologic surgery and breast plastic surgery.

Within these needs areas, the group discussed and agreed upon a range of initiatives and actions that the clinical community can take to improve both access to and outcomes from breast reconstructive surgery. These were as follows:

### Improved alignment and closer collaboration within a multidisciplinary approach

- The approach to breast cancer treatment should be based on a well-defined multidisciplinary team, including the relevant expertise to offer all surgical options.
- As local circumstances will vary, all institutions performing breast cancer surgery should develop an institution-specific guideline on the optimal patient pathway, incorporating all the relevant oncoplastic surgical expertise and the patient within the decision-making process.
- The institution-specific guideline should describe the entire treatment pathway and timetable, and define which clinical specialties should be involved at which points within that pathway. It is not possible to define a pathway that covers all eventualities for all patients, but the guideline should be applicable to the majority of surgical cases encountered.
- The institution-specific guideline should define the optimal time point for informing patients about their surgical and reconstructive options and provide a checklist of information that patients will be given, to allow them to make an educated decision.

### Surgical teamwork

- The initial decision on the type of cancer excision and reconstruction defines the end result.
- A good reconstruction begins with a good mastectomy.
- A successful breast conservation begins with a plan to restore/preserve the breast shape and symmetry.
- No single surgeon will be able to offer all oncoplastic treatment options and therefore a patient should be managed by a surgical team from the outset.
- Surgical teamwork is an integral part of the total treatment plan for any breast cancer treatment.

### Defining working-practice standards

The definition of working-practice standards is important in ensuring that institutions are working in a manner that optimizes patient care. The group identified a range of standards that should be applied to OPBS. These standards were defined at both institutional level and at the level of the individual surgeon:

Institutional standards

- Breast cancer surgery should be concentrated in institutions that can offer all of the possible tumor-excising and reconstructive options, and minimum standards should be set regarding which options those institutions offer.
- These standards should include measurable assessments of oncologic outcome (eg, margin involvement, local recurrence, survival), esthetic result, and patient satisfaction.
- Institutions should conduct a defined minimum number of procedures per year.^18^^,^^19^

Individual standards

- For the individual surgeon, measurable assessments of oncologic outcome, esthetic result, and patient satisfaction should be applied.
- Surgeons should belong to a multidisciplinary group responsible for institution-level breast cancer care.
- Surgeons should conduct (at least) a defined minimum number of oncoplastic breast surgery procedures per year.
- Surgeons should meet standards on complication rates.
- The aim is to accredit all oncoplastic breast surgeons using a set of minimum standards, according to local regulations.

## DISCUSSION

At present, the use of OPBS is suboptimal. This results from a lack of formal training for surgeons, a paucity of guidelines and inconsistent standards of care. This position paper represents a first step toward defining standards of care for breast reconstruction and providing guidance for the building of multidisciplinary teams with common goals in patient education, oncologic and esthetic outcomes, and patient satisfaction. The statements made here offer the perspectives of a group of European plastic surgeons on the key elements required for defining the optimal treatment pathway and standards of care for OPBS. We recognize that these statements set out an idealized position that may not be feasible in all institutions, in all countries, at the present time. However, if these goals can be achieved, outcomes for breast cancer patients are likely to be improved.
